# Supplementary material for: The effect of hydration status on plasma FGF21 concentrations in humans: A subanalysis of a randomised crossover trial
Source: PLoS One. 2020 Aug 5;15(8):e0235557. doi: 10.1371/journal.pone.0235557 (PMC7406048; doi:10.1371/journal.pone.0235557)
Supplement: Attachment — Submitted filename: Response to reviewers_June.docx [file pone.0235557.s003.docx]

We would like to thank once again the Editor and Reviewer for their review of our manuscript. We are pleased to read that our manuscript was reviewed overall favourably, with just minor comments to address. We hope we have addressed these adequately in the revised manuscript, with a full rebuttal below (in blue italics).

[Reviewer comment] Interesting sub analyis, although with some limitations. The present is a crossover RCT: this should be added in the title,. I would write; a sub analysis of a crossover RCT

*[Author response] Thank you for this helpful comment. We have changed the title to “The effect of hydration status on plasma FGF21 concentrations in humans: A subanalysis of a randomised crossover trial”.*

[Reviewer comment] Methods: tables should be put at end and not in the text

*[Author response] We followed PLoS author guidelines and inserted tables immediately after the paragraph they were first cited.*

[Reviewer comment] Methods: sample size calculation was not performed for this rct: this should be put in limitation.

*[Author response] Lines 264-270 discussed that the study was powered for glycaemia not FGF21. We have added the following (in red) to this paragraph to emphasise the point:*

*“It may be that there is a small but meaningful response, which we may not have been able to detect due to lack of statistical power for FGF21 as the outcome” (line 266)*

[Reviewer comment] Moreover this sub analysis was not prespecificed and also this should be added in limitation section

*[Author response] We hope it was clear this was not a pre-specified outcome in our introduction, but have reiterated this point in our discussion (addition in red):*

*“Nonetheless, future work should investigate the postprandial FGF21 response according to hydration status; this is particularly important considering our present analysis was decided post hoc and therefore not pre-specified in our research plan or trial registration.” (lines 256-258)*

Additional edit: Since resubmitting the manuscript, the first author has changed institutional affiliation, and this has been amended on the title page.

Our disclosures are also below as there was no place online to declare them, and the guidelines to authors stated to not put them in-text. We hope this is acceptable.

**Disclosures:** This project was supported by a graduate research grant from the European Hydration Institute, who had no involvement in the protocol design, data collection, analysis, or publications, and did not fund the FGF21 analysis. HAC has received conference fees, travel and accommodation (2016, 2017, 2018, and 2019) from Danone Nutriticia, has run a Tate and Lyle sponsored study, and has received funding from Esther Olssons II & Anna Jönssons Minnesfond foundations. J.A.B. has received funding from the BBSRC, GlaxoSmithKline, Lucozade Ribena Suntory, Kellogg’s, Nestlé and PepsiCo and is a scientific advisor to the International Life Sciences Institute (ILSI).
